# Limits of the Effective Medium Theory in Particle Amplified Surface Plasmon Resonance Spectroscopy Biosensors

**DOI:** 10.3390/s19030584

**Published:** 2019-01-30

**Authors:** Jefferson S. Costa, Quaid Zaman, Karlo Q. da Costa, Victor Dmitriev, Omar Pandoli, Giselle Fontes, Tommaso Del Rosso

**Affiliations:** 1 Department of Electrical Engineering, Federal University of Pará, Belém-PA 66075-110, Brazil; karlo@ufpa.br (K.Q.d.C.); victor@ufpa.br (V.D.); 2 Department of Physics, Pontifical Catholic University of Rio de Janeiro, Rio de Janeiro-RJ 22451-900, Brazil; quaid601@yahoo.com (Q.Z.); tommaso@puc-rio.br (T.D.R.); 3 Department of Chemistry, Pontifical Catholic University of Rio de Janeiro, Rio de Janeiro-RJ 22451-900, Brazil; omarpandoli@puc-rio.br; 4 Division of Metrology of Materials, National Institute of Metrology Quality and Technology - INMETRO, Duque de Caxias- RJ 25250-020, Brazil; gisellefontes@gmail.com

**Keywords:** Particle Amplified Surface Plasmon Resonance Spectroscopy (PA-SPR), Atomic Force Microscopy (AFM), Dielectric Loaded Waveguide (DLWG), Finite Element Method (FEM), Maxwell-Garnett effective medium theory

## Abstract

The resonant wave modes in monomodal and multimodal planar Surface Plasmon Resonance (SPR) sensors and their response to a bidimensional array of gold nanoparticles (AuNPs) are analyzed both theoretically and experimentally, to investigate the parameters that rule the correct nanoparticle counting in the emerging metal nanoparticle-amplified surface plasmon resonance (PA-SPR) spectroscopy. With numerical simulations based on the Finite Element Method (FEM), we evaluate the error performed in the determination of the surface density of nanoparticles *σ* when the Maxwell-Garnett effective medium theory is used for fast data processing of the SPR reflectivity curves upon nanoparticle detection. The deviation increases directly with the manifestations of non-negligible scattering cross-section of the single nanoparticle, dipole-dipole interactions between adjacent AuNPs and dipolar interactions with the metal substrate. Near field simulations show clearly the set-up of dipolar interactions when the dielectric thickness is smaller than 10 nm and confirm that the anomalous dispersion usually observed experimentally is due to the failure of the effective medium theories. Using citrate stabilized AuNPs with a nominal diameter of about 15 nm, we demonstrate experimentally that Dielectric Loaded Waveguides (DLWGs) can be used as accurate nanocounters in the range of surface density between 20 and 200 NP/µm^2^, opening the way to the use of PA-SPR spectroscopy on systems mimicking the physiological cell membranes on SiO_2_ supports.

## 1. Introduction

Surface plasmon resonance (SPR) spectroscopy, is today one of the principal optical techniques used for the development of low cost and high resolution chemical and bio-chemical optical sensors [1]. The detection principle of SPR sensors is based on the perturbation of the near electromagnetic field at the metal-dielectric interface of the sensing platform, provoked by the interaction of the external surface with the analytes of interest [2]. In order to enhance the sensitivity of the SPR-based optical sensors, different data processing methods and excitation or detection schemes have been proposed [3,4,5], and an ultimate SPR spectroscopy resolution of about 10^−7^ RIU has been determined in the classical Kreschtmann configuration with an angular interrogation scheme [6]. The latter value is associated to bulk refractive changes of the external medium. 

In some particular applications, the spatial overlap between the analytes and the evanescent electromagnetic field of the surface plasmon polariton (SPP) propagating along the metal-dielectric interface, has to be taken in account for the determination of the geometrical properties of the sensing platforms necessary to obtain the higher sensitivity. For example, when nanometric targets are considered, the best performances are obtained by the deposition of a ~30 nm thin film of SiO_2_ over the metal layer supporting the plasma wave [7]. Alternatively, the monitoring of the interaction of bio-molecules with systems mimicking the physiological cell membranes on solid supports is performed with the best sensitivity by the use of dielectric loaded waveguide (DLWG) [8,9], due to the wider extension of the evanescent electromagnetic field of the guided modes into the external medium. More recently, an alternative strategy consisting in the manipulation of the target species has been proposed for the enhancement of the sensitivity of the SPR optical sensor, giving rise to the so-called particle amplified surface plasmon resonance (PA-SPR) spectroscopy [10]. 

In PA-SPR spectroscopy, the analyte bio-molecules are previously chemically attached to the surface of gold nanoparticles (AuNPs), and the external surface of the sensing platform is functionalized with a proper bio-recognition element with high and selective affinity with the target bio-molecules. In the last decade, PA-SPR spectroscopy in the Kreschtmann configuration has demonstrated to enhance the sensitivity of optical bio-sensors up to 1000-fold enhancement [11,12,13,14]. Due to this extremely high sensitivity, PA-SPR imaging spectroscopy has also been recently applied for single nanoparticle detection, and a detailed study of the chemical affinity of different kinds of metal and dielectric nanoparticles with gold thin films modified by different ω-functionalized alkyl thiols [15]. 

In addition to the single nanoparticle detection [15], one of the challenges in PA-SPR spectroscopy consists in the fast measurement of the nanoparticle surface density σ, in order to monitor in real time the mass surface coverage of the target molecules which, linked to the AuNPs, interacts with the bio-recognition element deposited over the outer surface of the SPR sensing platform. The simplest approach for the fast and in real time determination of the mass surface coverage is the use of the Maxwell-Garnett (MG) theory which, due to the set-up of interparticle interaction, can be applied only in a definite range of σ. The limits of the effective medium theories in the interpretation of the experimental results in PA-SPR spectroscopy were investigated for the first time from an experimental point of view in [7,13]. In [13], the authors amplified the SPR signal by the use of biotin-capped AuNPs with a mean diameter of 24 nm, and observed that the increase in the surface density σ of the nanoparticles is associated to a progressive bigger deviation between the experimental and theoretical results, the latter obtained in the frame of the MG theory [16]. 

This discrepancy was associated to the onset of interparticle dipolar interactions, which are not the only cause leading to the failure of the effective medium theories in the determination of σ or, alternatively, of the effective dielectric function of the AuNPs/water composite layer. In fact, as pointed out experimentally in [7], when the distance between the gold layer supporting the plasma wave and the AuNPs gets progressively smaller, a distance-dependent intrinsic dielectric function of the nanoparticle has to be introduced to explain the experimental results. In the case of very thin SiO_2_ spacing layers (<10 nm), anomalous dispersion of the dielectric function of the AuNPs is observed [7], and numerical or semianalytical models taking into account the optical anisotropy of the AuNPs have to be used for the accurate determination of the surface density [17]. 

In the present paper, we perform a theoretical study of an SPR metal nanoparticle sensor in the Kreschtmann configuration, focused on the sensitivity response and the dependence of the dipole-dipole interactions on the dielectric spacer thickness, the dimension of the AuNPs, the surface density σ and the wavelength of the exciting electromagnetic wave. We compare a semi-analytical approach based on the effective medium theory, which neglects dipole-dipole interactions, with a numerical method developed by using Finite Element Method (FEM) in COMSOL Multiphysics® software [18], which gives exact results for the interpretation of the reflectivity curves of the sensors upon interaction with the AuNPs. 

The study defines the limited range of σ for which the counting of the nanoparticles interacting with the SiO_2_ surface of the sensing platform can be determined with a finite percentage accuracy *δσ* by the use of the effective medium theory. The importance of this research is not limited to PA-SPR spectroscopy, but extend to others amplified spectroscopies, such as the surface enhanced Raman spectroscopy (SERS) [19], where the knowledge of the surface density of nanoparticles is fundamental to measure the associated electromagnetic enhancement factor [19]. We conclude the article demonstrating for the first time experimental AuNP counting over DLWGs, presenting both TM_0_ and TM_1_ modes at the wavelength of 783 nm. In the low surface density regime, when dipole-dipole interparticle interactions are negligible, we show an excellent match between atomic force microscopy (AFM) and SPR-based results, demonstrating the possibility to extend in a near future the application of PA-SPR spectroscopy to the monitoring of nanoparticle uptake by lipidic or artificial cellular membranes [20,21].

## 2. Materials and Methods

### 2.1. Fabrication and Working Principle of the Au/SiO_2_ Sensing Platforms

**Figure 1:**
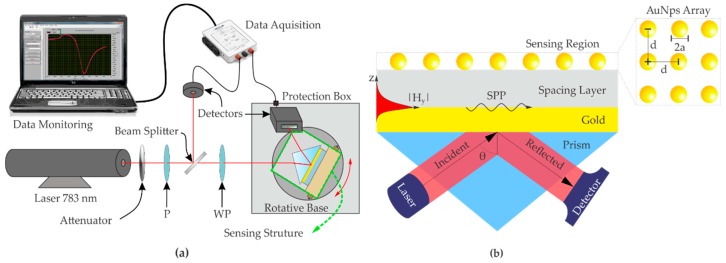
(**a**) Illustration of the experimental SPR set-up in Kreschtmann configuration. P: linear polarizer. WP: λ/4 wave-plate. (**b**) The sensing structure is composed of a coupling prism, a thin film of gold (~ 50 nm) and a dielectric spacer layer (SiO_2_).

The experimental set-up in the classical Kreschtmann configuration with angular modulation [2,22] and the Au/SiO_2_ sensing platforms fabricated in the present research, are represented in Figure 1a,b, respectively. In Figure 1a, a collimated laser source at the wavelength of 783 nm (model LM-783-PLR-75-1, 75 mW, Ondax Inc., Monrovia, CA, USA) impinges on the sensing platform after passing by a linear polarizer (P) and a λ/4 wave-plate (WP), used to select TM or TE polarization. The reflectivity of the SPR platform is measured in function of the angle of incidence *θ* (Figure 1b), using a controlled rotary base of Sigma-Koki with an angular resolution of 0.0025°. The reflectivity spectra are eventually sent to a data acquisition system connected to a personal computer (Figure 1a). When *θ* is greater than the attenuated total reflection angle (ATR) the SPP wave, here identified as TM_0_ mode, can be excited in TM polarization [23,24], and if the thickness of the SiO_2_ spacing layer is large enough, multiple resonances can be excited, both in TM and TE polarization [8,25]. 

An SF4 prism and a refractive index matching oil (*n* = 1.75, Cargille Laboratories, Cedar Grove, NJ, USA) was used for the optical coupling of the DLWGs, constituted by a thin film of gold of the thickness of about 49 nm and a 670 nm thick SiO_2_ film. 

The Au and SiO_2_ thin films were deposited by an electron beam assisted vacuum deposition system (Leybold, Univex 450, Colonia, Germany) at a pressure of about 3 × 10^−6^ Torr and with a rate of 3 Å/s. Prior to the deposition of SiO_2_, the gold-coated substrates were functionalized with 0.5% volume (3-mercaptopropyl) trimethoxysilane (MPTS) solution in ethanol (30 mL ethanol with 150 μL MPTMS) for 90 min at room temperature [22]. To carry out the hydrolysis process, the silanized gold film was immersed in a 0.1 M HCl water solution (0.85 mL of HCl in 100 mL of MilliQ water) for 6 h in room temperature. Finally, a condensation reaction is carried out at 60 °C for 3 h in oven. For the sensing of the AuNPs, the SiO_2_ layer was further functionalized by immersing the substrates in 3-aminopropyl trimethoxysilane (APTS)/ethanol solution for 2 h, followed by rinsing with ethanol and gentle drying with nitrogen [7]. 

**Figure 2:**
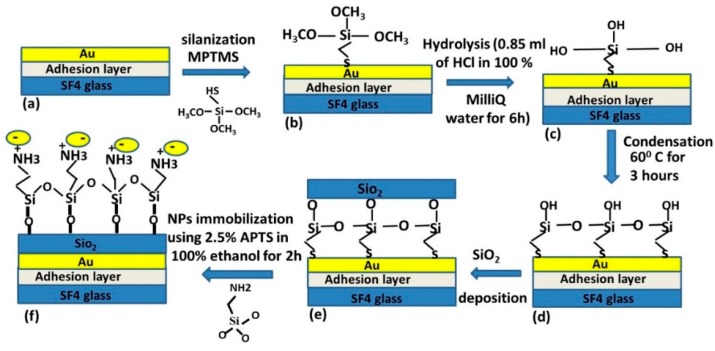
Schematic diagram showing the fabrication of the DLWGs. (**a**) 49 nm gold thin film deposited on clean SF4 glass. (**b**) Silanization with MPTS. (**c**) Hydrolysis process: methoxy groups are converted to Si-OH group. (**d**) Condensation leads to Si-O-Si network. (**e**) Deposition of SiO_2_ and (**f**) modification of SiO_2_ with amino group’s (-NH_2_) of APTS, which serves as molecular link for the negative charged citrate stabilized AuNPs.

Citrate stabilized AuNPs were synthesized using the procedure reported in [26]. A continuous flux pump (FutureChemistry, Nijmegen, The Netherlands), not shown in Figure 1, was used for sensor rinsing and nanomaterial injection. The composite layer consisting of AuNPs and H_2_O shown in Figure 1 is formed by fluxing citrate stabilized AuNPs at a rate flow 0.3 mL/min for times ranging from 1 to 5 min, to achieve different Nps surface density over the SiO_2_ surface. A schematic diagram showing the process of fabrication of the DLWGs, is represented in Figure 2.

In the theoretical calculations and in the interpretation of the experimental data we suppose the colloidal dispersion of AuNPs to be monodispersed, with all the nanoparticles having the same radius *a*. Moreover, the exact spatial distribution of the 2D array of AuNPs deposited over the SiO_2_ surface does not influence the optical response of the device. It depends only on the average surface density *σ* of the metal nanoparticles, validating the approximation of equispaced nanoparticles as shown in Figure 1a. 

When APTS molecules bind with AuNPs, the excitation condition of the wave modes in the sensor structure is altered with a final shift of the angle of resonance Δ*θ* [2]. The latter is used as the sensor output information and can be used to measure static parameters of the AuNPs thin film such as the electrical effective permittivity when the surface density is known experimentally [14,22]. Vice versa, the correct theoretical modeling of the dielectric constant of the AuNPs, allows the optical experimental measurement of the surface density of immobilized metal nanoparticles and, in this way, the calculus of the mass surface coverage of the target molecules along experiments in PA-SPR spectroscopy. 

### 2.2. Morphological Characterization of the DLWGs and AuNPs Citrate Colloidal Solution

**Figure 3:**
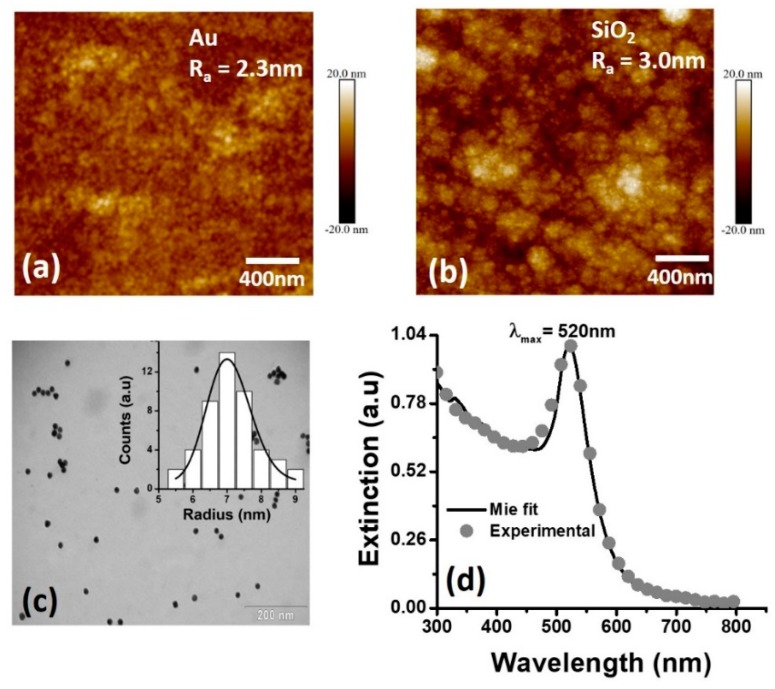
(**a**) AFM morphology image of the 49 nm gold thin film and (**b**) the 670 nm SiO_2_ layer constituting the DLWGs used in the present work. (**c**) Typical TEM image of the citrate colloidal solution of AuNPs used in the optical sensing experiments. A log-normal distribution is used to obtain the best fit to the experimental statistical distribution, represented as continuous line in the inset. (**d**) Comparison between the experimental (grey circles) and theoretical (continuous black line) extinction spectra of the citrate colloidal dispersion of AuNPs. The fit on the experimental data was obtained applying the Mie theory with quadrupole orders [31].

The surface of both the Au and SiO_2_ thin films constituting the DLWGs were analyzed by an AFM (model Multimode 8, Bruker, Santa Barbara, CA, USA), operated in Peak Force Tapping^TM^ with scanAssist Air tips (spring constant ≈ 0.4 N/m). Data analysis was carried out with Nanoscope Analysis software, version 1.4, by Bruker. The results are shown in Figure 3a,b. The route mean square (RMS) roughness of the surface increase from 2.3 nm in the case of bare gold to 3.0 nm after the deposition of the SiO_2_ thin film. These values are coherent with the typical values reported in literature for metallic thin films [27,28,29], and do not produce a significant damping of the SPPs propagating along the metal-dielectric interface [30]. 

The statistical size distribution of the colloidal solution of AuNPs was determined using a Tecnai Spirit Transmission Electron Microscope (FEI Company, model Tecnai Spirit G2, Hillsboro, OR, USA) operating at 30 kV with bright-field detector. The corresponding TEM image is shown in Figure 3c. In the inset, represented as a continuous line, the best fit on the experimental statistical distribution is shown. It was obtained by using a log-normal function [31]. 

From the fit on the experimental size distribution we obtained a mean radius value *<a>* = 7.3 nm, and a standard deviation *δ_α_* = 1.5 nm, representative of colloidal solutions with low polidispersitivity [32]. The size statistical distribution of Figure 3c is further used to perform the theoretical fit of Mie with quadrupole orders [31] on the experimental extinction spectra of the colloidal dispersion of AuNPs, as shown in Figure 3d. The matching between the experimental and the theoretical extinction spectra is excellent, thus supporting the reliability of the value of *<a>* obtained by TEM, which will be further considered as reference value. 

### 2.3. Theoretical Model

**Figure 4:**
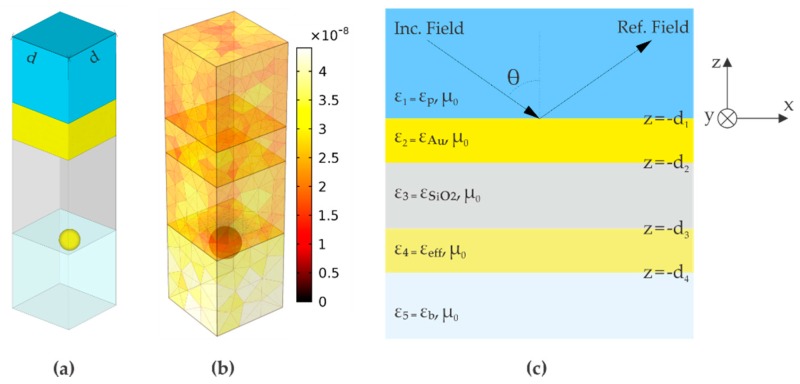
(**a**) Unit periodic cell considered in the numerical simulations by the COMSOL® software. The Floquet theorem is used to set the periodic boundary conditions. Periodic ports are used in the top boundary (set on to simulate the incident planar wave) and in the bottom boundary (set off to simulate a very large layer) [18]; (**b**) Mesh of the FEM model of the sensor. Colors indicate the size of the elements, which is defined by physical and geometrical factors; (**c**) Resultant planar structure of the PA-SPR sensor in the effective layer approximation to represent the AuNPs periodic array.

The numerical model in FEM of the SPR sensor (Figure 1) is based in a unit periodic cell of width *d*, containing one AuNP (Figure 4a). The mesh (Figure 4b) represents the division of the space in elements small enough to study correctly the propagation of the electromagnetic wave using the Radio Frequency (RF) module of COMSOL Multiphysics^®^ software [18]. In our calculations each material is characterized by its electrical permittivity. For the gold thin film, the dielectric constant is calculated using the Drude-Lorentz model with electron damping and one term of interband transitions [22,25]. 

The semi-analytical approach is based on the approximation of the composite thin film constituted by the external medium and the periodic array of AuNPs (Figure 1b) as an effective homogeneous layer with thickness *h_Np_* = 2*a* and effective permittivity *ε_eff_* (Figure 4c), given by the following Maxwell-Garnett (MG) mixing formula:

(1) $$\varepsilon_{eff}=\varepsilon_{b}\left[\frac{1+2f_{s}\left(\varepsilon_{Au}-\varepsilon_{b}\right)/\left(\varepsilon_{Au}+2\varepsilon_{b}\right)}{1-f_{s}\left(\varepsilon_{Au}-\varepsilon_{b}\right)/\left(\varepsilon_{Au}+2\varepsilon_{b}\right)}\right]$$

In the latter, *f_s_* = 2π/3(a/d)^2^ is the AuNPs volume fraction in the heterogeneous layer and *ε_b_* is the permittivity of the external medium. The surface density *σ* = 1/(*d*[µm])^2^ is measured in nanoparticles per µm^2^ (Np/µm^2^) [7,12,13,17]. The validity of equation (1) is limited by the onset of non-negligible scattering cross-section of the AuNPs, dipole-dipole interactions between adjacent nanoparticles and dipolar interaction between the nanoparticles and the metal thin film supporting the plasma wave [16,17,33].

The aim of the effective medium theory is to find the effective dielectric constant (1) of a composite layer constituted by nanosized inclusions contained in a non-absorbing dielectric matrix with the real dielectric constant *ε_b_*. In the present work, we need to model the composite AuNPs/water film as a continuous film, since the propagation of the incident plane wave in the resultant planar structure of the SPR sensor (Figure 4c) is analyzed by the generalized reflection coefficient $\tilde{r}$ for the planar multilayer structure [34]. Equation (2), representing the generalized reflection coefficient, is defined in frequency domain with the time dependence exp(–*iωt*), and *r* and *t* are the Fresnel’s reflection and transmission coefficients for TM or TE polarization in accordance with the excitation source:

(2) $${\tilde{r}}_{n,n+1}=\frac{r_{n,n+1}+{\tilde{r}}_{n+1,n+2}\exp\left[i2k_{n+1,z}\left(d_{n+1}-d_{n}\right)\right]}{1+r_{n,n+1}{\tilde{r}}_{n+1,n+2}\exp\left[i2k_{n+1,z}\left(d_{n+1}-d_{n}\right)\right]}$$

For TM polarization, the transverse magnetic field *H_n,y_* in the n-th layer of the sensor structure (Figure 4b) is given by equation (3), where *A_n_* is the amplitude of the field component given by equation (4). For TE polarization, equation (3) corresponds to the transverse electric field *E_n,y_* [34]. Equations (3) and (4) have the following form:

(3) $$H_{n,y}=A_{n}\left[\exp\left(-ik_{n,z}z\right)+{\tilde{r}}_{n,n+1}\exp\left[ik_{n,z}\left(2d_{n}+z\right)\right]\right]\exp\left(ik_{x}x\right)$$

(4) $$A_{n}=\frac{t_{n-1,n}A_{n-1}\exp\left[i\left(k_{n-1,z}-k_{n,z}\right)d_{n-1}\right]}{1-r_{n,n-1}{\tilde{r}}_{n,n+1}\exp\left[i2k_{n,z}\left(d_{n}-d_{n-1}\right)\right]}$$

In expressions (2)–(4) A_1_ = 1 is the amplitude of the incident field; $k_{n}^{2}=\omega^{2}\varepsilon_{n}\mu_{n}$ is the wave propagation constant in the n-th layer, *ω* = 2π*c/λ* is the angular frequency, *c* is the speed of light in free space, and *k_x_ = k*_1_ sin(θ) is the x-axis component of the wave vector. The reflectivity curve *R* is calculated by relation (2) considering the prism-gold interface, so that $R={\left|{\tilde{r}}_{12}\right|}^{2}$ [34]. Throughout the text, the bulk sensitivity *S* of the SPR sensing platform is defined as the ratio of the change in Δ*θ* to the change in the refractive index of the external medium *n_b_* [1,2,6,35,36,37]. 

## 3. Results and Discussion

### 3.1. Modal Analysis

**Figure 5:**
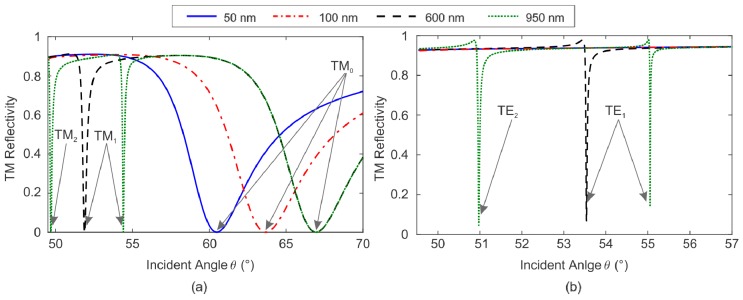
Reflectivity curves *R(θ)* at the wavelength of 633 nm with increasing values of *h_SiO2_* for both (**a**) TM and (**b**) TE polarizations. The arrows highlight the resonant wave mode for each minimum in *R*. The thickness of the gold thin film is *h_Au_* = 46 nm and the external medium is water.

The parameter *h_SiO2_* is crucial to study both modal characteristics and bulk sensitivity of the SPR platform sensing [7,8,9,11,36]. To achieve the *h_SiO2_* cutoff values defining the transition from pure plasmonic behavior to waveguide resonator, we show in Figure 5 the reflectivity curves *R(θ)* for *h_SiO2_* varying from 10 nm to 1000 nm, in both TM and TE polarizations, considering the exciting wavelength of 633 nm and water as the external medium. The resonance angle of the plasmonic mode (TM_0_) increases continuously with *h_SiO2_* up to saturation value of ~67°, for dielectric layer thicker than 300 nm (Figure 5a). Pockrand et al. first demonstrated this behavior [38]. The coupled waveguide modes TM_1_, TE_1_, TE_2_, and TM_2_ only raise for *h_SiO2_* thicker than the cutoff values of 240 nm, 400 nm, 770 nm, and 950 nm, respectively [8]. 

**Figure 6:**
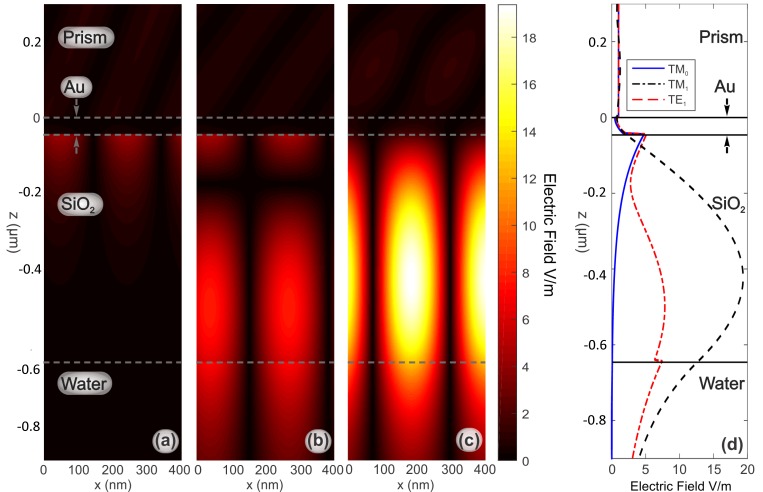
The absolute values of the electric field for the resonant modes in the SPR platform. In colors for a cut plane parallel to the zx-plane: (**a**) *E_z_* Field for TM_0_ mode with *θ* = 66.9°, (**b**) *E_z_* field for TM_1_ mode with *θ* = 51.88°, and (**c**) *E_y_* field for TE_1_ mode with *θ* = 53.57°; (**d**) Norm of E field for the resonant modes in a cut line parallel to the z-axis. Fixed parameters: λ = 633 nm, *h_Au_* = 46 nm, and *h_SiO2_* = 600 nm.

In Figure 6, we present the near electric field E in the resonant condition for the TM_0_, TM_1_ and TE_1_ modes when the dielectric thickness is 600 nm. The TM_1_ mode provides a better overlap between the E-field profile and the external environment if compared with the plasmonic mode, which leads to a higher sensitivity of the platform [35,36]. The TE_1_-field does not present an evanescent profile at the gold-dielectric interface as TM modes, but is characterized by a good overlap with the external environment, in agreement with [8].

**Figure 7:**
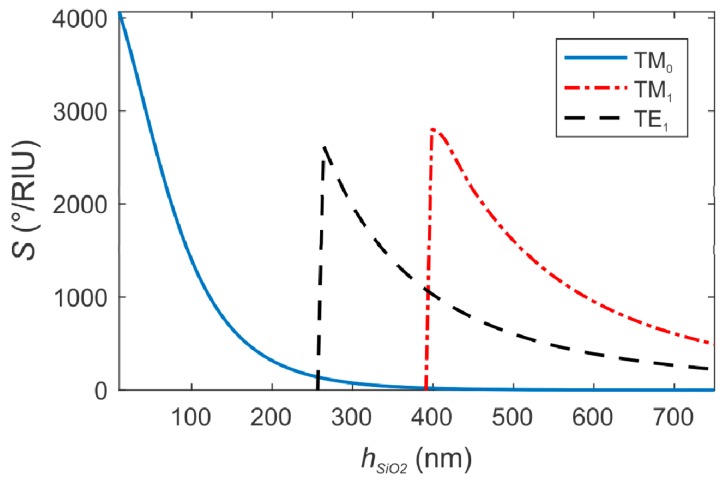
Bulk sensitivity S of the Au/SiO_2_ sensing platforms versus *h_SiO2_* for variation of the external refractive index from 1.33 to 1.36 RIU. Both plasmonic and DLWG regimes in TM and TE polarizations are considered.

In Figure 7, we represent the bulk sensitivity *S* (in °/RIU) of the Au/SiO_2_ sensing platforms versus *h_SiO2_* in the monomodal and DLWG regimes, for both TM and TE polarizations. For the TM_0_ mode, the sensitivity decreases to zero when *h_SiO2_* increases, with a maximum value of 4140 °/RIU for *h_SiO2_* = 10 nm. For the other modes of the DLWGs, the sensitivity also decreases with *h_SiO2_*, with maximum values of 2670 °/RIU for TM_1_ and 2560 °/RIU for TE_1_, occurring for thicknesses close to the corresponding cutoff values. 

Although the bulk sensitivity may be slightly different from the sensitivity of the sensor to a spatial confined material such as metal nanoparticles, the results shown in Figure 7 underline that for the multimodal DLWGs, the evanescent field associated to the TM_1_ mode has to be used as the electromagnetic sensing nanoprobes when the best sensitivity is desired.

### 3.2. Parametric Analysis of Models

**Figure 8:**
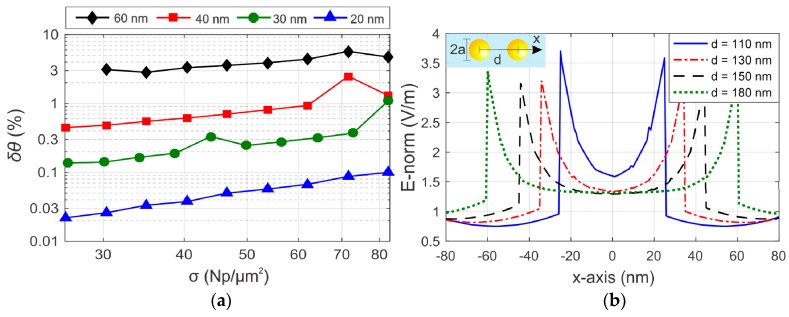
(**a**) Relative deviation *δθ* between the resonance angles *θ*_FEM_ and *θ*_MG_ relative to an Au/SiO_2_ monomodal sensing platform with a dielectric thickness of 50 nm. The data are represented for AuNPs diameters of 20, 30, 40 and 60 nm, and surface densities σ between 26 and 83 Np/µm^2^. (**b**) *E_z_* field between two adjacent AuNPs with the diameter of 60 nm embedded in water. The centers of the AuNPs are separated by the distance *d*, as highlighted in the inset.

To investigate the limits of the Maxwell-Garnet theory in the determination of the AuNPs surface density in PA-SPR spectroscopy, we evaluate the relative percentage deviation between the resonance angle of the SPR platforms calculated by the FEM method (*θ*_FEM_) and the semi-analytical model (*θ*_MG_). The difference $\delta \theta \left(\%\right)=100\%\left|\theta_{FEM}-\theta_{MG}\right|/\theta_{FEM}$ is calculated by probing the TM_0_ mode of an Au/SiO_2_ SPR platform with a dielectric thickness of 50 nm, considering an exciting wavelength of 633 nm and water as an external medium. In Figure 8a we can observe, from a general point of view, the tendency of the discrepancy *δθ* to get worse as *a* increases with a fixed value of *σ*, or when *σ* increases and the dimension of the AuNPs is fixed.

The tendency can be explained by two factors. The first one is the progressive deficiency of effective medium theories to model of the extinction properties of the single AuNP as the size increases, since the scattering properties are not taken in account in the Maxwell-Garnett theory [16]. The second one is the onset of dipole-dipole interaction between adjacent AuNPs when increasing the surface density *σ* or the single AuNP radius [16,17,33]. To show the influence of *σ* on the dipole-dipole interparticle interaction, we present in Figure 8b the near *E_z_* field in the center between two adjacent AuNPs with a diameter of 60 nm, separated by the distance *d*, at the wavelength of 633 nm. As a general trend, we observe an enhancement of the E-field between adjacent AuNPs as *d* decreases. Interestingly, for distances smaller than 130 nm (equivalent to *σ* ≈ 44 Np/µm^2^), it is observed a sudden increase in the field intensity in the middle of the AuNPs (point *x* = 0 nm in Figure 8b), due to onset of the dipole-dipole interparticle interaction regime [16]. 

**Figure 9:**
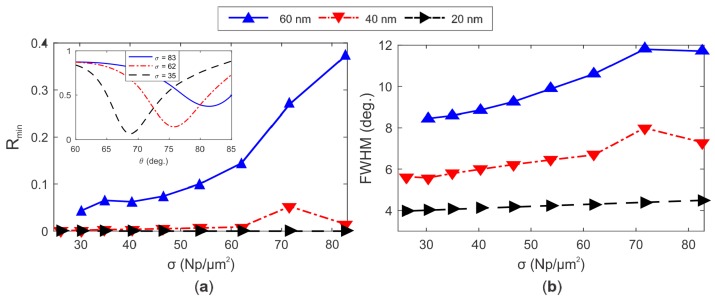
(**a**) Rmin and (**b**) FWHM of the reflectivity SPR spectra calculated by FEM for AuNPs with diameters of 60 nm, 40 nm, and 20 nm, and surface density in the range of 26 < *σ* < 83 Np/µm^2^. In (**a**), the inset highlights the reflectivity curves for 60 nm sized AuNPs and surface densities *σ* of 83, 62 and 35 Np/µm^2^.

The dipole-dipole interparticle interactions are also correlated with the increase of the minimum of reflectivity *R_min_* and the full width at half maximum (FWHM) of the SPR spectra. This is evident in the reflectivity curves of Figure 9, represented for AuNPs with a diameter of 60 nm, 40 nm and 20 nm. Particularly interesting, is the rapid increase of *R_min_* up to 0.38 for *σ* > 83 Np/µm^2^, which results from field overlap and dipolar interparticle interactions for the biggest nanoparticles [7,14,35]. 

In order to make quantitative and rapid measurements in PA-SPR spectroscopy, three conditions are highly desired: (i) the possibility to extend the surface density of the AuNPs array from low to high values (low *d*) in order to extend the maximum range of the sensor; (ii) the use of AuNPs with higher surface (big radius), in order to have the better results in the enhancement of the sensitivity compared to classical SPR spectroscopy in Kreschtmann configuration; and (iii) a fast calculus method based on the Maxwell-Garnet approximation.

**Figure 10:**
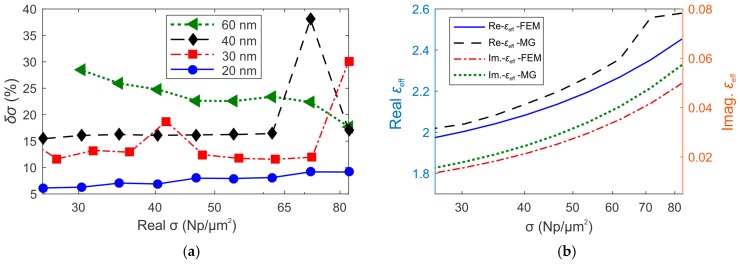
(**a**) The relative deviation δσ between the real surface density *σ*_FEM_ and the density *σ*_MG_ calculated applying the MG formula to fit the resonance angle *θ*_FEM_. (**b**) Comparison between the effective permittivity of the Au/water composite layer calculated using the MG mixing formula (MG-*ε*_eff_) and the correct real values obtained by FEM method (FEM-*ε*_eff_). The comparison has been performed considering AuNPs with a diameter of 40 nm.

On the basis of these conditions, the results of Figure 8 and Figure 9 suggest the use of an exciting radiation at higher wavelengths than 633 nm (near IR, i.e., 783 nm), since interparticle interactions are expected to rise at higher values of surface density in this case. A trade-off should be considered in the choice of both AuNP dimensions and maximum surface density of the array, depending on the error that can be tolerated in the evaluation of the surface density and, in ultimate analysis, in the calibration of the PA-SPR sensor. To define the error of the surface density *δσ*, we calculate the relative deviation between the real surface density *σ*_FEM_ used in FEM simulations, and the density calculated using MG formula (*σ*_MG_) to fit the TM_0_ resonance angle *θ*_FEM_ (Figure 10a), for the same AuNP size and surface density used in Figure 8a.

It is clear the increasing of the discrepancy δσ for higher values of the surface density (Figure 10a), especially for bigger nanoparticles. The best results are observed for AuNPs of 20 nm in diameter, for which the discrepancy remains lower than 10% for all the analyzed values of surface density. 

In Figure 10b, for each value of surface density *σ*_FEM_, we compare the effective permittivity of the AuNPs/water composite layer calculated by the use of the MG theory (MG-*ε_eff_*) and the values of *ε_eff_* calculated using the FEM method (FEM-*ε_eff_*). The calculations were performed considering AuNPs with a diameter of 40 nm. The FEM-*ε_eff_* values are larger than the MG-*ε_eff_* and the difference increases with *σ*. Again, this particular behavior is associated to the progressive contribution of the interparticle dipolar interactions for higher values of surface density.

**Figure 11:**
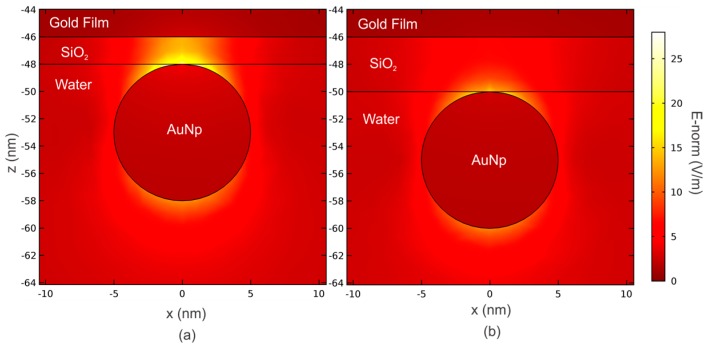
Norm of the near electric field surrounding the AuNPs for thin dielectric spacer layers: (**a**) *h_SiO2_* = 2 nm and (**b**) *h_SiO2_* = 4 nm. The parameters are *λ* = 633 nm, *h_Au_* = 46 nm, *a* = 5 nm, *σ* = 40 Np/µm^2^, and *θ* = 60°.

Besides of the limitations of the MG theory due to the onset of the interparticle interactions, it is important to highlight the effects of dipolar interaction between the AuNPs and the gold thin film supporting the plasma wave in the presence of thin dielectric layer spacer [7,12,13]. In Figure 11, we illustrate the near E-field surrounding a single AuNP with a diameter of 10 nm with an excitation wavelength of 633 nm. In this case, we consider a value of *σ* low enough to disregard the dipole-dipole interaction between the AuNPs, and a nanoparticle size small enough to validate the quasi-static regime [16]. The intensity of the electromagnetic field at the SiO_2_/Au interface clearly shows the onset of AuNP-Au thin film for very small values of *h_SiO2_* [7,17,39]. 

**Figure 12:**
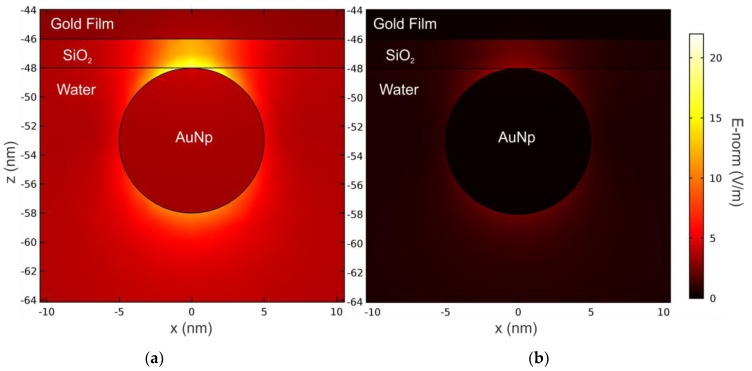
Norm of the near electric field surrounding an AuNP for a thin dielectric spacer layer with a thickness of 2 nm, calculated at the wavelengths of (**a**) 633 nm and (**b**) 783 nm. Fixed parameters: *h_Au_* = 46 nm, 2*a* = 10 nm, *σ* = 40 NP/µm^2^, and *θ* = 60°.

The AuNP-Au thin film interaction is also dependent on the wavelength [7,13,17,39]. Figure 12 shows the near-field surrounding a 10 nm-AuNP over a 2 nm thick SiO_2_ layer at the wavelengths of 633 nm and 783 nm. In this case, we observe that the interaction decreases with the wavelength, in accordance with [39]. 

**Figure 13:**
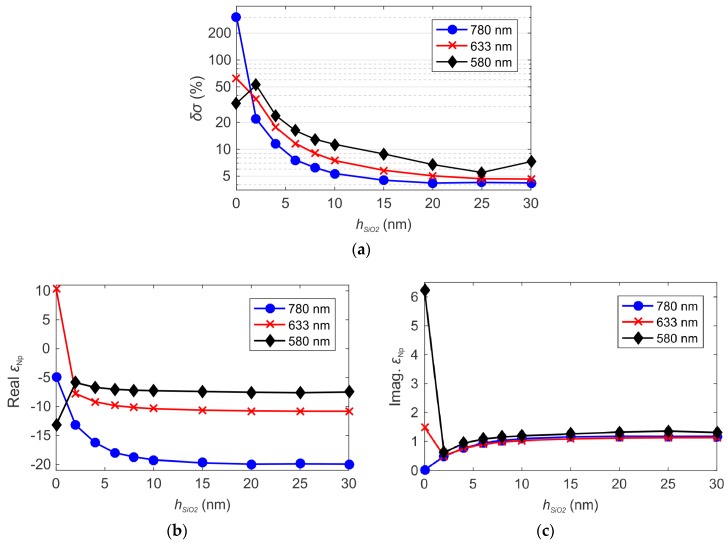
(**a**) Relative percentage deviation δσ between the real surface density *σ_FEM_* and the density *σ_MG_* calculated using the MG theory to fit the TM_0_ resonance angle *θ_FEM_*. Real (**b**) and imaginary (**c**) parts of the dielectric constant of the AuNPs (*ε_Np_*) calculated by the MG theory in order to fit the exact reflectivity curves calculated by the FEM method. The results are shown in function of *h_SiO2_* and for the excitation wavelengths of 580 nm, 633 nm and 780 nm. Fixed parameters: *h_Au_* = 46 nm, 2*a* = 10 nm and *σ* ≈ 40 NP/µm^2^.

To investigate the limits of the MG theory due to the AuNP-Au thin film interaction, we calculated the percentage discrepancy *δσ* between *σ*_FEM_ and *σ*_MG_ depending on the SiO_2_ thin film thickness. The results, reported in Figure 13a, were obtained for three different excitation wavelengths. We observed that, for the same wavelength, the discrepancy *δσ* reduces increasing the thickness of the SiO_2_ layer. Vice versa, for the same value of *h_SiO2_*, *δσ* decreases for higher excitation wavelengths. These general tendencies in the behavior of δσ can be explained with the set-up of the dipolar interaction between the single isolated AuNP and the thin Au layer constituting the Au/SiO_2_ sensing platform [7,39], coherently with the results reported in Figure 11 and Figure 12. In our calculation for the 10 nm sized AuNPs, we observed that δσ is maintained below 10% for all the excitation wavelengths considered, when the SiO_2_ thickness is higher than 20 nm. 

In Figure 13b,c we compare the real and imaginary parts of the dielectric constant of the single AuNP (*ε_Np_*) calculated by the MG theory in order to fit the exact reflectivity curves calculated by the FEM method, as proposed in [7]. Interestingly, when the dielectric thickness is smaller than ~ 10 nm, we observe an expressive variation in *ε_Np_*, which induces to a wrong interpretation of the experimental results, such as anomalous dispersion in the real part of the dielectric constant of the nanoparticles, as observed in [7]. Here, we show clearly by the near field simulation reported in Figure 12, that the anomalous dispersion is effectively due to the onset of AuNP-Au thin film dipolar interaction, which cannot be taken in account by the use of the classical effective medium theories [16]. 

### 3.3. Experimental SPR AuNPs Counting

The DLWGs fabricated in the present research were tested as nanoparticle counter for AuNPs with the nominal diameter of 14.6 nm, a dimension for which the Maxwell-Garnett theory can be still applied under the assumption of inclusions with negligible value of the scattering-cross section [40]. Following the theoretical results reported in Section 3.1, the TM_1_ mode was used as the most sensitive optical nanoprobe for the detection of the AuNPs in DLWG regime. 

The performances of the DLWGs in NP counting were studied by SPR spectroscopy and AFM in a range of σ between ~20 and 200 NP/µm^2^, in order to prevent the effects of dipole-dipole interparticle interaction as highlighted in Section 3.2. Prior to the optical sensing experiments, the optical constants and thickness of the gold and SiO_2_ layers constituting the DLWGs were characterized by two-color SPR spectroscopy using the experimental procedure reported in [41]. In Table 1, the values obtained for the parameters of the DLWGs at the experimental wavelength of 783 nm are shown.

**Figure 14:**
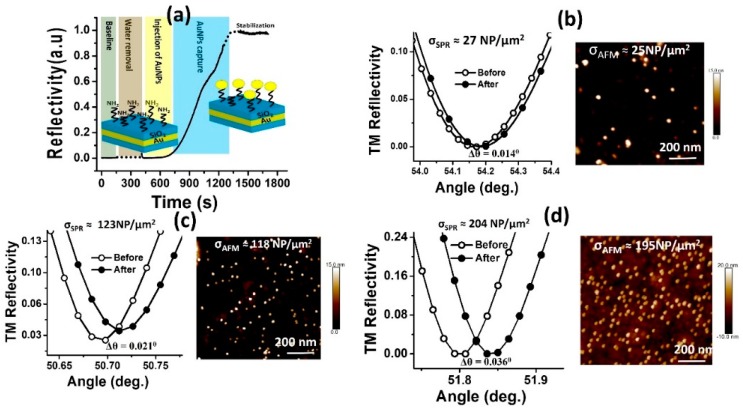
Principle of work of SPR nanoparticle counting with DLWGs. (**a**) SPR sensorgram relative to the sensing of the interaction between the amino group NH^2+^ of the external surface of the DLWGs and the negatively charged AuNPs. The TM_1_ mode of the DLWG has been used as evanescent optical probe. The excitation wavelength was 783 nm, and the sensorgram was taken at a fixed incidence angle of 50.695°. (**b**) Comparison between experimental *σ*_SPR_ and *σ*_AFM_ in the non-interacting surface density regime: experimental SPR reflectivity curve of the TM1 mode of the DLWGs in water before and after interaction of the AuNPs with the SiO_2_ surface (left side), and AFM image of a 1 µm × 1 µm region of the SiO_2_ surface of the device analyzed by SPR spectroscopy (right size), with a surface density of 25 NP/µm^2^. (**c**) *σ* = 120 NP/µm^2^. (**d**) *σ* = 200 NP/µm^2^. The average surface density *σ*_AFM_ was calculated by analysis of four different regions of each sample.

In Figure 14a, the time-dependent SPR sensorgram relative to the different experimental phases involved the sensing of the AuNPs is reported. The different experimental phases consist in base-line determination for TM1 mode in a pure water environment, water removal with enhancement of the reflectivity (not shown), injection of the colloidal solution of nanoparticles with monitoring of the interaction between the amino group of the SAM of APTMS and the negative charge of the AuNPs, and final pure water rinsing and stabilization of the SPR signal. The raise of the reflectivity is due to a shift Δ*θ* in the SPR angle after AuNPs detection and stabilization. In Figure 14b–d, the shift of the SPR reflectivity curves and AFM image of the external surface of the DLWGs after nanoparticles sensing with a final surface density of about 25, 120 and 200 NP/µm^2^ are shown.

As it is shown in the AFM images reported in Figure 14, the AuNPs are randomly distributed on the surface, which may not follow the simulation modeling proposed in Section 2 based on an ordered bidimensional array of AuNPs. This doesn´t represent a crucial issue when the metal nanoparticles are not interacting one with the other. In the approximation of non-interacting nanoparticles, the exact spatial distribution of the bidimensional array of AuNPs deposited over the SiO_2_ thin film of the sensor does not influence the optical response of the device, and the shift in the angle of resonance of the DLWGs upon the interaction with the AuNPs depends only on the surface density *σ*, and not on their specific surface distribution. 

This assumption is validated by the excellent matching between the AFM and SPR experimental densities *σ*_SPR_ and *σ*_AFM_ during the nanoparticle counting in the range between approximately 20 and 200 NPs/µm^2^. Progressive increasing surface density will lead to partial agglomeration of the AuNPs, breaking the approximation of non-interacting nanoparticles for values of surface density lower than the ones predicted by the theoretical analysis. Anyway, the value of surface density at which the AuNPs starts to agglomerate and the non-interacting nanoparticle approximation starts to fail may depend in principle on several physico-chemical factors [15] such as the dimension of the nanoparticle, the surface density of the molecular ligands on the SiO_2_ surface of the waveguide, the flux velocity of the AuNPs during the injection process, or the pH of the water environment containing the colloidal dispersion of AuNPs. The optimal choice of the experimental parameters during the metal nanoparticle deposition to prevent their agglomeration and extend the range of the sensor, represent an interesting physico-chemical investigation which we pretend to address in the near future. 

## 4. Conclusions

In this paper, we analyzed in details the limits of the Maxwell-Garnett theory in the determination of the surface density σ of AuNPs in PA-SPR spectroscopy. Using the Kreschtmann configuration and an Au/SiO_2_ sensing platform, we compared the changes in the reflectivity curves of the SPR sensor upon interaction with a bidimensional array of spherical AuNPs, applying mathematical models based on FEM and MG theory. 

We observed a progressive decrease in the accuracy of the optical predictions of the effective medium theory when increasing either the surface density or the size of the AuNPs. The failure of the MG theory is attributed to the onset of dipolar interparticle interaction between adjacent AuNPs, as clearly observed by optical near-field simulations performed by FEM. The theoretical results show that the discrepancy in the determination of σ remains below 10% for surface densities of the order of ~100 Np/µm^2^, when small nanoparticles with a diameter of about 20 nm are considered.

Our calculations by the FEM, also showed that the accuracy of the MG theory in the prediction of the optical properties of the AuNP/water thin film decreases with the onset of the AuNP-Au thin film interaction, and confirm that the anomalous dispersion usually observed experimentally when very thin dielectric thin films are used, is due to the failure of the effective medium theories to take in account this particular dipolar interaction. The error in the estimation of σ by the use of the MG theory is anyway maintained below 10% when dielectric spacers with a thickness higher than about 20 nm are considered.

We conclude that three conditions are necessary for a quantitative, rapid and accurate measurements of σ in an extended range: (i) the introduction of a SiO_2_ spacing layer with a thickness higher than ~20 nm; (ii) the use of AuNPs with a diameter smaller than ~20 nm; (iii) the use of excitation wavelengths in the near infrared region, in order to have a reduced interparticle interaction for high values of σ.

On these bases, we demonstrated experimentally the functionality of the Maxwell-Garnett theory for the accurate counting of small AuNPs (2*a* = 14.6 nm) deposited on DLWGs. The experiments were successfully conducted in the range between 20 and 200 NP/µm^2^, demonstrating an excellent matching between atomic force microscopy (AFM)- and SPR-based results in nanoparticle counting. The results suggest that in the case of small AuNPs, the DLWGs might be successfully used for quantitative measurements of *σ* in PA-SPR spectroscopy, paving the way to perform quantitative measurement of nanoparticle uptake by lipidic or artificial cellular membranes [43].

## Figures and Tables

**Table 1:** The thickness and dielectric constants of the gold thin film and SiO_2_ obtained from the SPR curves fitting.

λ (nm)	Gold		SiO_2_		Water
783	*h_Au_* (nm)	*ε =* *ε_r_ + i* *ε_i_*	*h_SiO2_* (nm)	*ε_r_*	*ε_r_*
	44.57	−23.91 + i1.72	644	2.11	1.76 [42]
